# Head repositioning errors in normal student volunteers: a possible tool to assess the neck's neuromuscular system

**DOI:** 10.1186/1746-1340-14-5

**Published:** 2006-03-06

**Authors:** Edward F Owens, Charles NR Henderson, M Ram Gudavalli, Joel G Pickar

**Affiliations:** 1 Associate Professor, Palmer Center for Chiropractic Research, 741 Brady Street, Davenport, IA 52803, USA; 2 Professor, Palmer Center for Chiropractic Research, 741 Brady Street, Davenport, IA 52803, USA; 3 Adjunct Associate Professor, Dept of Biomedical Engineering, University of Iowa, Iowa City, IA 52240, USA

## Abstract

**Background:**

A challenge for practitioners using spinal manipulation is identifying when an intervention is required. It has been recognized that joint pain can interfere with the ability to position body parts accurately and that the recent history of muscle contraction can play a part in that interference. In this study, we tested whether repositioning errors could be induced in a normal population by contraction or shortening of the neck muscles.

**Methods:**

In the experimental protocol, volunteers free of neck problems first found a comfortable neutral head posture with eyes closed. They deconditioned their cervical muscles by moving their heads 5 times in either flexion/extension or lateral flexion and then attempted to return to the same starting position. Two conditioning sequences were interspersed within the task: hold the head in an extended or laterally flexed position for 10 seconds; or hold a 70% maximum voluntary contraction in the same position for 10 seconds. A computer-interfaced electrogoniometer was used to measure head position while a force transducer coupled to an auditory alarm signaled the force of isometric contraction. The difference between the initial and final head orientation was calculated in 3 orthogonal planes. Analysis of variance (1-way ANOVA) with a blocking factor (participants) was used to detect differences in proprioceptive error among the conditioning sequences while controlling for variation between participants.

**Results:**

Forty-eight chiropractic students participated: 36 males and 12 females, aged 28.2 ± 4.8 yrs. During the neck extension test, actively contracting the posterior neck muscles evoked an undershoot of the target position by 2.1° (p <0.001). No differences in repositioning were found during the lateral flexion test.

**Conclusion:**

The results suggest that the recent history of cervical paraspinal muscle contraction can influence head repositioning in flexion/extension. To our knowledge this is the first time that muscle mechanical history has been shown to influence proprioceptive accuracy in the necks of humans. This finding may be used to elucidate the mechanism behind repositioning errors seen in people with neck pain and could guide development of a clinical test for involvement of paraspinal muscles in cervical pain and dysfunction.

## Introduction

An important consideration for practitioners using spinal manipulation is knowing when to intervene (i.e., determining the presence of a manipulable lesion). A number of local measures have been used to identify a dysfunctional segment, including: tissue compliance, static and motion palpation, x-ray, surface EMG, and thermography. Global measures have also been used to determine the region of the affected segment(s), such as leg length inequality, sacro-occipital technique tests, and visual inspection of posture (reviewed in [1]). Because no gold standard exists for the presence of a manipulable lesion, the validity of many of these measures is unknown.

We are interested in determining if a proprioceptive test could be applied to the neck that might serve as a global measure of neuromuscular function and reveal differences between normal subjects and those who respond to spinal manipulation. As a first step toward this goal and as described in this report, we sought to determine in a relatively normal student population whether repositioning errors of the neck could be induced based upon the thixotropic properties of muscle spindles.

Thixotropic properties of skeletal muscle were first described by Hill [2] for extrafusal muscle fibers where a slow lengthening evokes a rapid rise in passive muscle tension that subsequently falls and plateaus to a constant level of passive tension. Hill termed the marked muscle stiffness at the beginning of the slow lengthening as a short-range elastic component (SREC). The SREC was attributed to spontaneous formation of actin-myosin crossbridges in the extrafusal fibers of passive muscle, crossbridges that form within several seconds of holding the muscle at a fixed length prior to the slow lengthening [2]. These crossbridges are relatively stable, having a slower turnover rate compared with crossbridges that underlie active muscle contraction.

Many studies also support the presence of the SREC in intrafusal fibers, the effect of which alters the responsiveness of Group Ia and II spindle afferents whose receptive endings are wound around the intrafusal fibers. Muscle history induced by maintaining intrafusal fibers at a shortened length, either by passive shortening, by active extrafusal muscle contraction, or by nerve stimulation sufficient to activate gamma-motoneurons increases muscle spindle responsiveness compared with not having previously shortened the intrafusal fibers [3-6]. The shortened intrafusal fibers are thought to crosslink and stiffen at the short length (see [7-9] for extensive discussions) and, with subsequent stretch, these stiffened myofilaments deform the receptive endings to a greater extent. On the other hand, maintaining intrafusal fibers at a long length stiffens the spindle apparatus at the longer length. As the muscle is subsequently returned to a shorter length, the stiffened intrafusal myofilaments become slack or kink and the receptive endings are unloaded. When the extrafusal muscle is stretched again, these slack intrafusal fibers are not initially loaded and hence the onset of spindle activity is delayed and spindle response is depressed.

Previous studies in humans and cats demonstrate that muscle history affects muscle spindle discharge resulting in proprioceptive consequences including alterations in spindle mediated muscle reflexes and errors in limb repositioning [3-5,10-14]. In cats, isometric contraction of the soleus muscle at a short length, as compared to isometric contraction at a long length, increases muscle spindle discharge to muscle stretch from identical intermediate positions [4]. In the lumbar spine of the cat, segmental changes in vertebral position can affect muscle history and the responsiveness of lumbar paraspinal muscle spindles [15,16]. In cats and humans, muscle history affects the magnitude of the stretch reflex and, at the same time, produces converse effects on the H-reflex arising from changes in resting spindle discharge [12,14]. Additionally, actively contracting a shortened biceps brachii muscle leads to errors in forearm position in humans [4]. To our knowledge, the effects of muscle history in the cervical spine on errors in repositioning are unknown.

Our specific aim was to determine if the mechanical history of cervical paraspinal muscles affects an asymptomatic individual's ability to reposition his/her cervical spine. We tested the following hypotheses: 1) when participants passively hold their necks in an extended or laterally flexed position for 10 seconds, they will demonstrate a repositioning error that undershoots the target position more than if they had not extended or laterally flexed their neck; 2) active contraction of the cervical muscles to 70% of their maximum voluntary contraction (MVC) while holding the neck extended or laterally flexed for 10 seconds produces an even greater repositioning error than seen with passive extension or lateral flexion.

## Methods

Both male and female volunteers were sought among the student population of a chiropractic college. Participants were included if they were between the ages of 20 and 40 yrs, had no recent incidence of cervical pain or trauma, showed no abnormalities on screening cervical x-rays, lacked cervical tenderness or muscle spasm with palpation, and had no gross limits upon cervical range of motion examination. Participants accepted into the study were shown a short video of the procedure, signed an informed consent form approved by the Institutional Review Board, and were randomly assigned to one of twelve different testing sequences that randomized the presentation order of experimental interventions.

**Figure 1:**
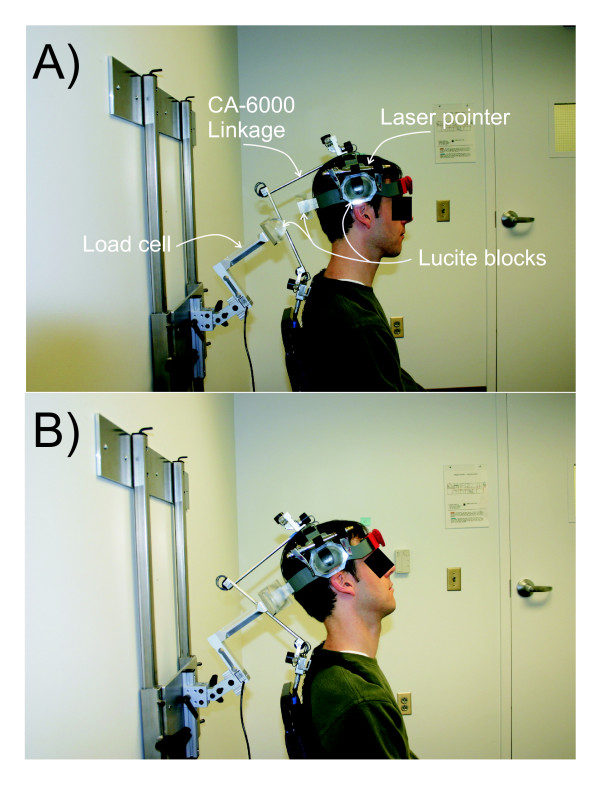
Photographs of the experimental equipment. A) a participant in the neutral position in preparation for an Extension test. B) a participant in the extended position. The CA-6000 linkage measures head position relative to the base affixed at the first thoracic vertebra. Matching Lucite blocks, one attached to the headband of the CA-6000, and the other attached to the load cell, provide for alignment during the neck extension. In this position the patient can exert force against the load cell for measuring maximum voluntary contraction during the "Active Hold" conditioning.

Figure 1 shows the features of the experimental apparatus. A computer interfaced electrogoniometer (CA-6000, OSI Corporation, Union City, CA) was used to measure head position and motion with respect to the upper thoracic spine in the 3 cardinal planes: sagittal (AP-flexion), frontal (lateral flexion), and horizontal (rotation). The CA-6000 headpiece was fitted with a laser pointer to help participants relocate their neutral head position between protocols. A force transducer (ESP-55, Transducer Techniques, Temecula, CA) was used to measure the force of an MVC by the neck muscles against a reference docking station. Mated pieces of a machined Lucite block were mounted to the CA-6000 headpiece and to the docking station, thereby providing a reference stop position and stabilizing the participant's head at the limit of movement.

Each test began by having a participant locate a comfortable, neutral head position. Participants were instructed to close their eyes, nod a few times and then return their head to a comfortable resting position. This became the neutral target position and was identified by marking the projection of the laser light on a large screen located 6' in front of the participant. The inclination of the headpiece with respect to vertical was recorded and MVC was determined separately with the neck extended 20° or left laterally flexed 25°. The examiner encouraged the participant to contract into the docking station as much as possible in order to achieve an MVC.

The experimental protocol began by having participants locate their previously established neutral target position by aligning the laser light with the screen mark. Participants maintained the neutral target position for 10 seconds with eyes open and were then instructed to close their eyes for the remainder of the protocol. Participants maintained the neutral position for an additional 10 seconds with eyes closed. Head position was recorded with the CA-6000 using a sampling frequency of 100 Hz. Participants deconditioned their neck muscles by performing five 20° neck extensions or five 25° left lateral flexions (hereafter referred to as lateral flexion) while the examiner coached the participant to maintain a steady cadence (0.75 cycle/sec using metronome feedback). Subsequently, one of three conditioning sequences was performed.

1) "No Hold" conditioning: participants immediately repositioned their heads to their perceived neutral target position.

2) "Passive Hold" conditioning: participants extended their necks 20° or laterally flexed them 25° and passively maintained that position for 10 seconds.

3) "Active Hold" conditioning: identical to Passive Hold, except that participants contracted their neck muscles for 10 seconds at the 20° extension or 25° lateral flexion position producing at least 70% MVC.

The participant's 70% MVC was signaled by a programmable process meter with an audio alarm (DP25-E, Omega Engineering, Inc., Stamford, CT) attached to the docking station. Following each conditioning sequence, participants attempted to reposition to the neutral target position. Data was collected for 10 seconds while participants maintained their heads in the perceived target position, still with eyes closed. Neck extension and lateral flexion tests were accomplished in random order. The 3 conditioning sequences were also performed in consecutive but random order, yielding a randomized complete block design.

**Figure 2:**
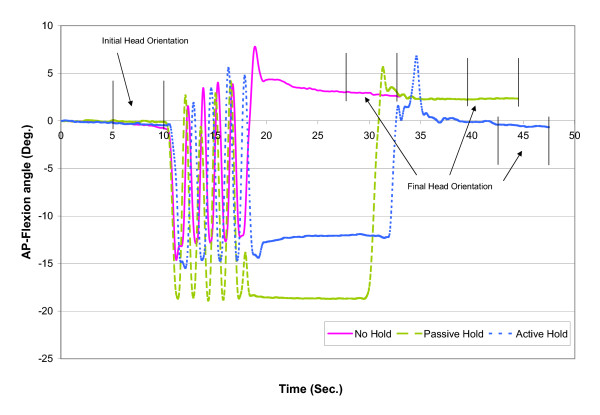
A plot of raw AP-Flexion motion in the Extension test for one participant. The sequence of activities is evident: 10 seconds of static neutral posture at the initial target position, followed by 5 deconditioning repetitions of neck extension. In the "No Hold" condition, the patient attempts to retarget to neutral immediately. In both "Passive Hold" and "Active Hold," there is a 10-second delay in the extended position. Vertical lines indicate the 5-second intervals over which average values were obtained for initial and final head orientation. In this particular case, both the No Hold and Passive Hold conditions produced an overshoot of the target position by 2.5 degrees. The Active Hold condition actually produced more accurate repositioning.

Data were exported as text files and reduced using custom software written in MathCad (Version 11, Mathsoft Inc, Cambridge, MA). Variables of primary interest were the average head orientation at the target position for 5 seconds before deconditioning and the average head orientation at the target position for 5 seconds after conditioning (see vertical lines in Figure 2 depicting the time intervals). The difference between the initial and final orientation was calculated in the 3 orthogonal planes and used as a measure of proprioceptive error. Negative values indicated repositioning that undershot the target position and conversely, positive values indicated repositioning that overshot the target position. Analysis of variance (1-way ANOVA) with a block factor (participants) was used to detect differences in proprioceptive error among the 3 conditioning sequences while controlling for variation between participants. Six ANOVA tests were performed to evaluate proprioceptive errors in 3 cardinal plane motions for the two tests (neck extension and lateral flexion), but were not corrected for multiple testing. However, when the F-test yielded significance, we performed post-hoc tests using Hochberg correction for multiple pairwise comparisons, alpha level 0.05.

## Results

Forty-eight students participated, 36 males and 12 females. Ages ranged from 21–40 yrs (mean ± SD = 28.2 ± 4.8 yrs). Extension MVC magnitudes ranged from 55.0 – 194.8 N (113.5 ± 35.2 N) and lateral flexion MVC ranged from 33.0 – 177.2 N (85.7 ± 31.4 N). Figure 2 shows a typical plot of the AP-flexion raw data for one participant during the extension test.

During the neck extension test, No Hold and Passive Hold conditioning sequences evoked AP flexion (sagittal plane) overshoots of the neutral target position that were not statistically different from each other (0.72° and 0.75°, respectively, Table 1). By contrast, the Active Hold conditioning sequence evoked an undershoot of the target position (-1.40°, Table 1) that was statistically significant when compared with No Hold and Passive Hold conditioning. This represented a 2.1° difference evoked by Active Hold conditioning. Repositioning in the frontal and horizontal planes showed no dependence on the conditioning sequence during the extension test.

During the lateral flexion test, the 3 types of conditioning sequences produced no differences in repositioning to the neutral target within the same plane as the test, i.e., within the frontal plane consisting of lateral flexion motion (p = 0.109). However, for orientation within the sagittal plane, Active Hold conditioning produced an AP flexion overshoot (2.01°, Table 1) that was significantly greater than that observed in No Hold conditioning. Repositioning in the horizontal plane showed no dependence on the conditioning sequence during the lateral flexion test.

## Discussion

The aim of the present study was to determine if proprioceptive errors based upon cervical paraspinal muscle history could be measured in normal subjects. We hypothesized, based upon a thixotropic mechanism (described in the Introduction), that participants who maintained their cervical muscles passively shortened for 10 seconds would demonstrate a repositioning error that undershot the target position and, in addition, that active muscle contraction with the muscles at the same shortened length would produce an even greater repositioning error.

The results of the extension test in our study are consistent with a thixotropic mechanism and support our main thesis that the recent history of cervical paraspinal muscle contraction accompanied by muscle shortening affects the ability of participants to accurately reposition their heads to a neutral head position. We found a statistically significant difference in repositioning error in AP-flexion during the extension task when the posterior muscles were contracted with the head in an extended position. The 70% MVC used for the active contraction likely recruited a substantial number of gamma-motoneurons because gamma-motoneurons are coactivated with alpha-motoneurons even at a low level of force [17]. Hutton et al. [18] found that muscle history-induced repositioning errors of elbow flexors were graded with the percent MVC and that the errors were greatest when voluntary muscle contraction was maximal.

We expected the Passive Hold conditioning sequence to produce a repositioning error similar in direction, but of lesser magnitude than the Active Hold conditioning. Our data, however, showed the Passive Hold repositioning error to be similar in magnitude to the No Hold control. The 20° neck extension we used to passively shortening the posterior neck muscles may have been of insufficient magnitude to adequately shorten the intrafusal fibers.

It was curious that the lateral flexion test was not similarly affected by the Active Hold condition. We expected to see a repositioning error in the main plane of repositioning motion (i.e. lateral bending in the frontal plane, see Table 1). Instead, there was an overshoot in an orthogonal plane (i.e. AP-flexion in the sagittal plane) that was significantly different from the No Hold condition. We think this may have been a consequence of our experimental setup. Participants often tucked their chins in order to seat the mating pieces of the Lucite block. Hence, the conditioning in lateral flexion was not a pure motion, but frequently was accompanied by anteroflexion. The deep anterior cervical flexors likely contracted, which could have produced the AP flexion overshoot – just the converse to the AP flexion undershoot seen in the extension test. A future study employing electromyography might help resolve this issue. The finding that repositioning errors can be seen in motions orthogonal to the main testing motion underscores the need for observing all planes of motion during both the conditioning and repositioning tasks.

Head repositioning tasks are not solely dependent on proprioceptive input from muscle, but also depend on visual and vestibular input. With multiple trials a learning effect may also occur. We removed contributions from the visual system by having participants close their eyes throughout the test. Differential effects of vestibular inputs were minimized because head positions were identical during passive and active conditioning (dictated by the shape of the fixed Lucite block, see Figure 1) and because the durations of passive and active conditioning were identical. The systematic effects of memory or learning were minimized in that conditioning tasks were presented in random order. Their potential contributions should have been distributed equally across the conditioning protocols. Thus, it seems a reasonable conclusion that the repositioning errors we measured arose from the effects of muscle history we engendered.

Repositioning in the cervical spine has been used to assess neck function. Several studies have examined the potential of repositioning errors after head movement for detecting abnormalities in the neck. Revel et al [19] asked blindfolded patients to reposition their heads to a target position subsequent to maximal unilateral rotation. Individuals with cervical pain did not reposition their heads as well as individuals without cervical pain. Revel et al [19] noted a consistent overshoot, which he attributed to a search for additional proprioceptive information. In other studies, active head repositioning to a target position is impaired in individuals with whiplash [20] and dizziness of suspected cervical origin [21], but not in individuals with non-traumatic neck pain [22]. Several new approaches have been used to present more involved challenges to the neck's proprioceptive system in an effort to provide a diagnostic or prognostic tool [23,24]. Interestingly, these studies suggest that methods involving complex movements or novel starting positions show poor reproducibility, while more simple tests that attempt to relocate the neutral head position, as was done in the present study, are more accurate and reproducible.

The approach presented in this study may provide an evaluative tool to investigate the neck proprioceptive system and thereby identify neuromuscular dysfunction and/or its response to treatment. Since we were able to elicit a repositioning error in normal, healthy student volunteers, it raises the question of whether patients with neck problems, especially of non-traumatic origin, express repositioning errors different from non-symptomatic controls. Perhaps patients with vertebral fixation or relative segmental inflexibility identified as subluxations by chiropractors and somatic dysfunction by osteopaths are more or less prone to the effects of muscle thixotropy. For example, some neck conditions are already accompanied by muscle contraction or increased muscle tone which might occlude the effects we observed with Active Hold conditioning or reveal effects from Passive Hold conditioning. The clinical utility of this head repositioning test can be provided by comparing a population of non-symptomatic participants with others showing some clinical signs of joint dysfunction in the neck.

## Conclusion

The goal of this project was to investigate the possible use of a muscle-based proprioceptive task as an evaluative tool in the cervical spine. In normal subjects we found a statistically significant difference in repositioning error in AP-flexion during the extension task after isometric muscle contraction for 10 seconds, suggesting that the recent history of cervical paraspinal muscle contraction can influence the ability to accurately reposition the head. The condition of muscle shortening by resting the head in an extended position for 10 seconds did not show a different repositioning error from control. To our knowledge this is the first time that muscle mechanical history has been shown to influence proprioceptive accuracy in the necks of humans. This finding may be used to elucidate the mechanism behind repositioning errors seen in people with neck pain. We suggest that a clinical test might be developed using a reposition task with active and passive conditioning to test the involvement of paraspinal muscles in cervical pain and dysfunction.

## Declaration of competing interests

The author(s) declare that they have no competing interests.

## Authors' contributions

JP initiated the study. JP, CH and MG contributed to study design and equipment construction. EO coordinated the project including volunteer recruitment and data collection. EO JP and MG handled the data analysis. JP and EO wrote the first manuscript draft and all authors read and approved the final manuscript.

## Figures and Tables

**Table 1:** Proprioceptive error calculated as the difference between the average initial reference position and the position on retargeting after the conditioning sequence.

TEST	Cardinal Plane (Motion)	n	Mean proprioceptive error by conditioning sequence. In degrees ± SD			Statistic (p)
				
			No Hold	Passive Hold	Active Hold	
EXTENSION	Sagittal Plane (AP-Flexion)	48	0.72 ± 2.61	0.75 ± 3.28	-1.40* ± 3.29	F_2,94 _= 8.85 (<0.001)
	Frontal Plane (Lateral Bending)	45	0.05 ± 1.21	-0.20 ± 1.49	-0.01 ± 1.73	F_2,88 _= 0.54 (0.59)
	Horizontal Plane (Rotation)	48	0.12 ± 1.57	-0.10 ± 1.26	-0.05 ± 2.09	F_2,94 _= 0.40 (0.67)

LATERAL FLEXION	Sagittal Plane (AP-Flexion)	48	0.31 ± 2.44	0.93 ± 2.94	2.02^† ^± 3.30	F_2,94 _= 6.70 (0.002)
	Frontal Plane (Lateral Bending)	45	0.09 ± 1.76	-0.07 ± 2.45	0.79 ± 2.37	F_2,88 _= 2.27 (0.11)
	Horizontal Plane (Rotation)	48	1.01 ± 1.50	1.18 ± 1.92	1.17 ± 2.26	F_2,94 _= 0.15 (0.86)

* Significantly different from 2 other conditioning sequences in post-hoc analysis (p < 0.002)

^† ^Significantly different from No Hold conditioning sequence in post-hoc analysis (p < 0.002)
